# Supplementary material for: Rapid turnover of life-cycle-related genes in the brown algae
Source: Genome Biol. 2019 Feb 14;20:35. doi: 10.1186/s13059-019-1630-6 (PMC6374913; doi:10.1186/s13059-019-1630-6)
Supplement: Supplementary file 3 — Protein alignments, corresponding Gblocks html file and CDS sequences for the lineage conserved gametophyte-biased genes. (ZIP 2109 kb) [file 13059_2019_1630_MOESM3_ESM.zip › Con_GA_alignments/Ec-18_002320.aa_ali.fasta-gb.htm]

Ec-18\_002320.aa.fasta.trans.aa\_ali.fasta


## Gblocks 0.91b Results

Processed file: **Ec-18\_002320.aa.fasta.trans.aa\_ali.fasta**  
Number of sequences: **2**  
Alignment assumed to be: **Protein**  
New number of positions: **441** (selected positions are underlined in blue)

```
                         10        20        30        40        50        60
                 =========+=========+=========+=========+=========+=========+
Ec-18_002320     MRWGHSLHTVTREAMEEPWRGDPTRRGRGPAALVTVRHPERKHGRERHQGGRQGGKGSTL
MSTRG.39253.1    ------------------------------------------------------------
                                                                             


                         70        80        90       100       110       120
                 =========+=========+=========+=========+=========+=========+
Ec-18_002320     LRTAPEVLPDAAAAAAAMKLSVAPAHGGVGDTATGQPVSREMKGSAGTAKVAGRVVTGKG
MSTRG.39253.1    ----------------------AVATAALNGKAVGQ-TTAEAAGA---A--GGRLV-RKG
                                       ##############                        


                        130       140       150       160       170       180
                 =========+=========+=========+=========+=========+=========+
Ec-18_002320     RAGCGSRVARLGSALDNVETRADEARGRSGRGVRASSGQRLNLPCTKRHVKIVRAITVGD
MSTRG.39253.1    RS-MGDRVARLGLALDDAETRADRTGGKASKGLRARSGQRLNLPCAKRHIKIIRSMTLGD
                     ########################################################


                        190       200       210       220       230       240
                 =========+=========+=========+=========+=========+=========+
Ec-18_002320     ASAAAGNG--VGGVGGGGGTRGTNGSGPYPCTPKSS-------SGGKQVNTISPSPALSQ
MSTRG.39253.1    AGAAAGNVTPGGGVIGAYASVGAGSTGPNGLNTKTNGLRGPSGVGGKSAGAARALPASQS
                 #######    #######################          ################


                        250       260       270       280       290       300
                 =========+=========+=========+=========+=========+=========+
Ec-18_002320     A-------------------TATAATAAPPPSPVSAGVGTAVAVAPIEGAVSLRTVGKTV
MSTRG.39253.1    AGGSATAAAAAAGHPDLRVTVDSATLVPAAAAAATATSATAAGATPVEGAVSLKTIGKTV
                 #                                  #########################


                        310       320       330       340       350       360
                 =========+=========+=========+=========+=========+=========+
Ec-18_002320     AFLANLASPSAAGRDGDGGVFGGGGKTAGVSGTGSAYGPRQCWYSAAAAKQISDEAHEGQ
MSTRG.39253.1    AFLAKFAAPANEGRDADGGAFGGGGKMAGGGGGRSSYGLRQSWYSAAAAKAISEEAPEEG
                 ############################################################


                        370       380       390       400       410       420
                 =========+=========+=========+=========+=========+=========+
Ec-18_002320     DSAEEEGQAALELERRLHAPPAIQFVTETRRGGFNKGSTITYGSTITLQAFHGGILCFNN
MSTRG.39253.1    GDLEDEGEAALALERLLHAPPALQFVSESRRGGFNKGSTITYGSTITLQAFHGGILCFNG
                 ############################################################


                        430       440       450       460       470       480
                 =========+=========+=========+=========+=========+=========+
Ec-18_002320     KGKAQASALGAQPNALLTVWNLQDLQSKGPVRYGDVLLLQMGRHEVMGSSLIMHKEPETP
MSTRG.39253.1    KGKAQASALGAQPNALLTVWNLQDLQSKGPVRYGDVLFLQMGRHEVMGSSLIMHKDPKQK
                 #########################################################   


                        490       500       510       520       530       540
                 =========+=========+=========+=========+=========+=========+
Ec-18_002320     GSTHSSKRSGGVGSQASAGGVTGEQQTEDATRNRTSAG-SRAGPSKSSSGGSGGSA-GGG
MSTRG.39253.1    EGAQ---SSAGAGSALSV-GTSGEQETEEAHRGRASTTAGRAGSSKGSSGGSGGGAAPGS
                                    #################    ################  ##


                        550       560       570       580       590       600
                 =========+=========+=========+=========+=========+=========+
Ec-18_002320     VGAAVGNPVAVPFRGKNMDKAKHTGRWVIFHKDDPEATQGSEVCHLDRVVLMQTWLSLAS
MSTRG.39253.1    AGVAVGNAVAVPFRGKNMEKAKHTGRWVIFHKDEPEATQGSEVCHLDKVVLMQTWLSLAS
                 ############################################################


                        610       620       630       640       650       660
                 =========+=========+=========+=========+=========+=========+
Ec-18_002320     DRPADACLKQCGSVDDHCSRQQHQSRSSTAGRERSDGGVSGRGGGGRTGARGDGAGEQRG
MSTRG.39253.1    DRPSAACLKQSGSVDDYCYRPQLMRTSTKP------------------------------
                 ###########################                                 


                        670       680       690       700       710       720
                 =========+=========+=========+=========+=========+=========+
Ec-18_002320     SRQDVVVAKSVFAVRPECGWVISGVEDSGSGKRTGGGGGAGGRSSSATLLEGAATRQLRR
MSTRG.39253.1    ------------------------------------------------------------
                                                                             


                        730       740       750       760       770       780
                 =========+=========+=========+=========+=========+=========+
Ec-18_002320     SKSARLITATSVILQMRSNGQSIVRRAQEIFDETQRRRLACLRQKLLRTFRRERSRNWLP
MSTRG.39253.1    ------------------------------------------------------------
                                                                             


                        790       800       810       820       830       840
                 =========+=========+=========+=========+=========+=========+
Ec-18_002320     KRGGDRQLSIVYGPSSPWGKREALRLQREQHKQFEKVTAKAKIVGDIRNPTPFEVTRAEN
MSTRG.39253.1    --------------------------------------------------T---------
                                                                             


                        850       860       870       880       890       900
                 =========+=========+=========+=========+=========+=========+
Ec-18_002320     GALATEAKVKQEVWDALLKRYGYQEVTDMLALMHSASVIRRAVRAMRQRKWTRKMAKIDP
MSTRG.39253.1    ------------------------------------------------------------
                                                                             


                        910       920       930       940       950       960
                 =========+=========+=========+=========+=========+=========+
Ec-18_002320     EMVEKVERLRKIQEQRKAKMNGGGVSATSSGRGVDSAPSVTNGEDEPRDDHAIAATGPAS
MSTRG.39253.1    ------------------------------------------------------------
                                                                             


                        970       980       990      1000      1010      1020
                 =========+=========+=========+=========+=========+=========+
Ec-18_002320     AGTAGAAAAAAVAAPSAPASVTATADRGAQRPSRPYEALLAGSLEKFEGDHRHGHGGGDG
MSTRG.39253.1    ------------------------------------------------------------
                                                                             


                       1030      1040      1050      1060      1070      1080
                 =========+=========+=========+=========+=========+=========+
Ec-18_002320     GGSGDATAKASGGSLTRGARGGRPLSSGGKPRFNTIGGRGKDAEREESCREKERQTRAKA
MSTRG.39253.1    ------------------------------------------------------------
                                                                             


                       1090      1100      1110      1120      1130      1140
                 =========+=========+=========+=========+=========+=========+
Ec-18_002320     RPKTAGEGSGGVQGVLARMGGYEKSVFREFGGDGRGGAVDQQLSRDVVLLEDLRGTLTQV
MSTRG.39253.1    ----------GIDGV---------------------------------------------
                                                                             


                       1150      1160      1170      1180      1190      1200
                 =========+=========+=========+=========+=========+=========+
Ec-18_002320     MAWDLTQGGAGTDPAAAGSVAAGHGTGGNTQEKGVSPDSSLVDESPVAKNHHAASRLSGV
MSTRG.39253.1    ------------------------------------------------------------
                                                                             


                       1210      1220      1230      1240      1250      1260
                 =========+=========+=========+=========+=========+=========+
Ec-18_002320     AAVKTNGKDRRASASSSGLEVSERNVGGAGDENLVEHVPVTLAGHHVDGSDEFLRHTGED
MSTRG.39253.1    ------------------------------------------------------------
                                                                             


                       1270      1280      1290      1300      1310      1320
                 =========+=========+=========+=========+=========+=========+
Ec-18_002320     PGASPTATRQRSACPRGRRVVGGGGSGAAEDANWSASTSDDAGSTSTAASAASARSCKRR
MSTRG.39253.1    -----------------RR--GGSG--SGD------------------------------
                                                                             


                       1330      1340      1350      1360      1370      1380
                 =========+=========+=========+=========+=========+=========+
Ec-18_002320     TPPAPLTDDERMSMLQHIRPSVFGEPTAFSQRRRAAVTAARPRSAAAGAVEHSGHRRGIP
MSTRG.39253.1    ------------------------------------------------------------
                                                                             


                       1390      1400      1410      1420      1430      1440
                 =========+=========+=========+=========+=========+=========+
Ec-18_002320     GGNHHQPPRASTARRTSRRGESDSVYRGSGIGAPKHRGILVHDGWVAGQGEPSSCAFSTS
MSTRG.39253.1    ------------------------------------------------------------
                                                                             


                       1450
                 =========+=========
Ec-18_002320     TGATPASESRPRSLTCRRL
MSTRG.39253.1    -------------------
```

```
Parameters used
Minimum Number Of Sequences For A Conserved Position: 2
Minimum Number Of Sequences For A Flanking Position: 2
Maximum Number Of Contiguous Nonconserved Positions: 8
Minimum Length Of A Block: 10
Allowed Gap Positions: None
Use Similarity Matrices: Yes
```

```
Flank positions of the 8 selected block(s)
Flanks: [83  96]  [125  187]  [192  214]  [225  241]  [276  477]  [500  516]  [521  536]  [539  627]  

New number of positions in Ec-18_002320.aa.fasta.trans.aa_ali.fasta-gb:  441  (30% of the original 1459 positions)
```
